# Copper(II) and silver(I)-1,10-phenanthroline-5,6-dione complexes interact with double-stranded DNA: further evidence of their apparent multi-modal activity towards *Pseudomonas aeruginosa*

**DOI:** 10.1007/s00775-021-01922-3

**Published:** 2022-01-10

**Authors:** Anna Clara Milesi Galdino, Lívia Viganor, Matheus Mendonça Pereira, Michael Devereux, Malachy McCann, Marta Helena Branquinha, Zara Molphy, Sinéad O’Carroll, Conor Bain, Georgia Menounou, Andrew Kellett, André Luis Souza dos Santos

**Affiliations:** 1 Department of General Microbiology, Institute of Microbiology Paulo de Góes, Universidade Federal do Rio de Janeiro, Rio de Janeiro, Brazil; 2 Institute of Chemistry, Postgraduate Program in Biochemistry, Universidade Federal do Rio de Janeiro, Rio de Janeiro, Brazil; 3 The Centre for Biomimetic and Therapeutic Research, Focas Research Institute, Technological University Dublin, Dublin, Ireland; 4 CICECO—Aveiro Institute of Materials, Department of Chemistry, University of Aveiro, Aveiro, Portugal; 5 Chemistry Department, Maynooth University, Kildare, Ireland; 6 School of Chemical Sciences and The National Institute for Cellular Biotechnology, Dublin City University, Dublin, Ireland; 7 SSPC, The SFI Research Centre for Pharmaceuticals, School of Chemical Sciences, Dublin City University, Glasnevin, Dublin 9, Ireland

**Keywords:** *Pseudomonas aeruginosa*, Coordination compounds, Antimicrobial action, DNA binding, DNA oxidative damage, Mechanism of action

## Abstract

Tackling microbial resistance requires continuous efforts for the development of new molecules with novel mechanisms of action and potent antimicrobial activity. Our group has previously identified metal-based compounds, [Ag(1,10-phenanthroline-5,6-dione)_2_]ClO_4_ (Ag-phendione) and [Cu(1,10-phenanthroline-5,6-dione)_3_](ClO_4_)_2_.4H_2_O (Cu-phendione), with efficient antimicrobial action against multidrug-resistant species. Herein, we investigated the ability of Ag-phendione and Cu-phendione to bind with double-stranded DNA using a combination of in silico and in vitro approaches. Molecular docking revealed that both phendione derivatives can interact with the DNA by hydrogen bonding, hydrophobic and electrostatic interactions. Cu-phendione exhibited the highest binding affinity to either major (− 7.9 kcal/mol) or minor (− 7.2 kcal/mol) DNA grooves. In vitro competitive quenching assays involving duplex DNA with Hoechst 33258 or ethidium bromide demonstrated that Ag-phendione and Cu-phendione preferentially bind DNA in the minor grooves. The competitive ethidium bromide displacement technique revealed Cu-phendione has a higher binding affinity to DNA (*K*_app_ = 2.55 × 10^6^ M^−1^) than Ag-phendione (*K*_app_ = 2.79 × 10^5^ M^−1^) and phendione (*K*_app_ = 1.33 × 10^5^ M^−1^). Cu-phendione induced topoisomerase I-mediated DNA relaxation of supercoiled plasmid DNA. Moreover, Cu-phendione was able to induce oxidative DNA injuries with the addition of free radical scavengers inhibiting DNA damage. Ag-phendione and Cu-phendione avidly displaced propidium iodide bound to DNA in permeabilized *Pseudomonas aeruginosa* cells in a dose-dependent manner as judged by flow cytometry. The treatment of *P. aeruginosa* with bactericidal concentrations of Cu-phendione (15 µM) induced DNA fragmentation as visualized by either agarose gel or TUNEL assays. Altogether, these results highlight a possible novel DNA-targeted mechanism by which phendione-containing complexes, in part, elicit toxicity toward the multidrug-resistant pathogen *P. aeruginosa*.

**Graphical abstract:**

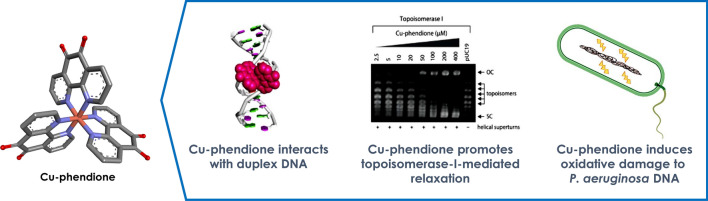

**Supplementary Information:**

The online version contains supplementary material available at 10.1007/s00775-021-01922-3.

## Introduction

The therapeutic application of metal-based complexes has emerged against a multitude of human pathological disorders [1, 3]. These treatments range from cisplatin in antineoplastic chemotherapy, gold-coordinated compounds for slowing the progression of rheumatoid arthritis, bismuth-based drugs for the treatment of ulcers, antimony-based metallodrugs in antiparasitic therapy, and silver-containing compounds with antimicrobial action [1–4].

1,10-Phenanthroline (1,10-phen) is a promising ligand in the development of new metal-based compounds [5, 6]. The rigid structure of the aromatic rings of 1,10-phen facilitates the formation of stable complexes with metal ions, thereby enabling the synthesis of a wide variety of coordination compounds [7, 8]. Moreover, the extension of the 1,10-phen backbone at the -5,6-position allows for efficient modulation of its antimicrobial action [6, 9]. With the addition of an *o*-quinoid group at the -5,6-position on the 1,10-phen backbone, 1,10-phenanthroline-5,6-dione (phendione) has exhibited increased antimicrobial activity when compared to 1,10-phen [10, 11]. Phendione-based complexes have demonstrated excellent antiproliferative activity against the: metronidazole-resistant *Trichomonas vaginalis* [12], dematiaceous fungus *Phialophora verrucosa* [13], clinically relevant yeast *Candida albicans* [14, 15], multidrug-resistant strains of *Candida haemulonii* species complex [16], filamentous fungus *Scedosporium apiospermum* [17], *Escherichia coli* [18], methicillin-resistant *Staphylococcus aureus* [18], carbapenemase-producing *Acinetobacter baumannii* [19], and multidrug-resistant bacterium *Pseudomonas aeruginosa* [9].

In general, both 1,10-phen- and phendione-based complexes can interact with DNA by semi-intercalating or electrostatically binding in the minor groove. Several of these complexes can induce DNA damage by cleaving DNA [20–23]; however, they may also cause indirect injuries through structural distortion thereby affecting the machinery that maintains DNA integrity [24, 25]. Gopu et al. [26] showed that (BOPIP = {2-(4-(benzyloxy)phenyl)-1H-imidazo[4,5-f]1,10-phen}) and its mono-nuclear Ru(II) polypyridyl complexes exhibited a significant antiproliferative activity against human tumor cell lines (A549, Du145, HeLa), as well as inhibiting the growth of *E. coli* and *S. aureus*. The formation of DNA adducts with Ru(II)-complexes and the DNA damage induced by these compounds play a significant role in their anticancer and antimicrobial activity [26]. The antimicrobial action of lanthanide phendione-based complexes, ([Eu(TFN)_3_(phendione)], [Eu(HFT)_3_(phendione)] and [Yb(HFA)_3_(phendione)]), against *E. coli, S. aureus* and *Proteus penneri* were recently correlated to their binding to the bacterial DNA [27].

The current study first aimed to investigate whether [Cu(phendione)_3_](ClO_4_)_2_.4H_2_O and [Ag(phendione)_2_]ClO_4_ were able to interact with double-stranded DNA using in silico and in vitro approaches. The ability of these test compounds to cleave DNA and to inhibit topoisomerase I activity was also evaluated. Finally, we investigated the possible interaction of both complexes with *P. aeruginosa* chromosomal DNA which could explain, at least in part, the antimicrobial action recently identified against this clinically relevant human pathogen [11].

## Materials and methods

### Test compounds

**Fig. 1:**
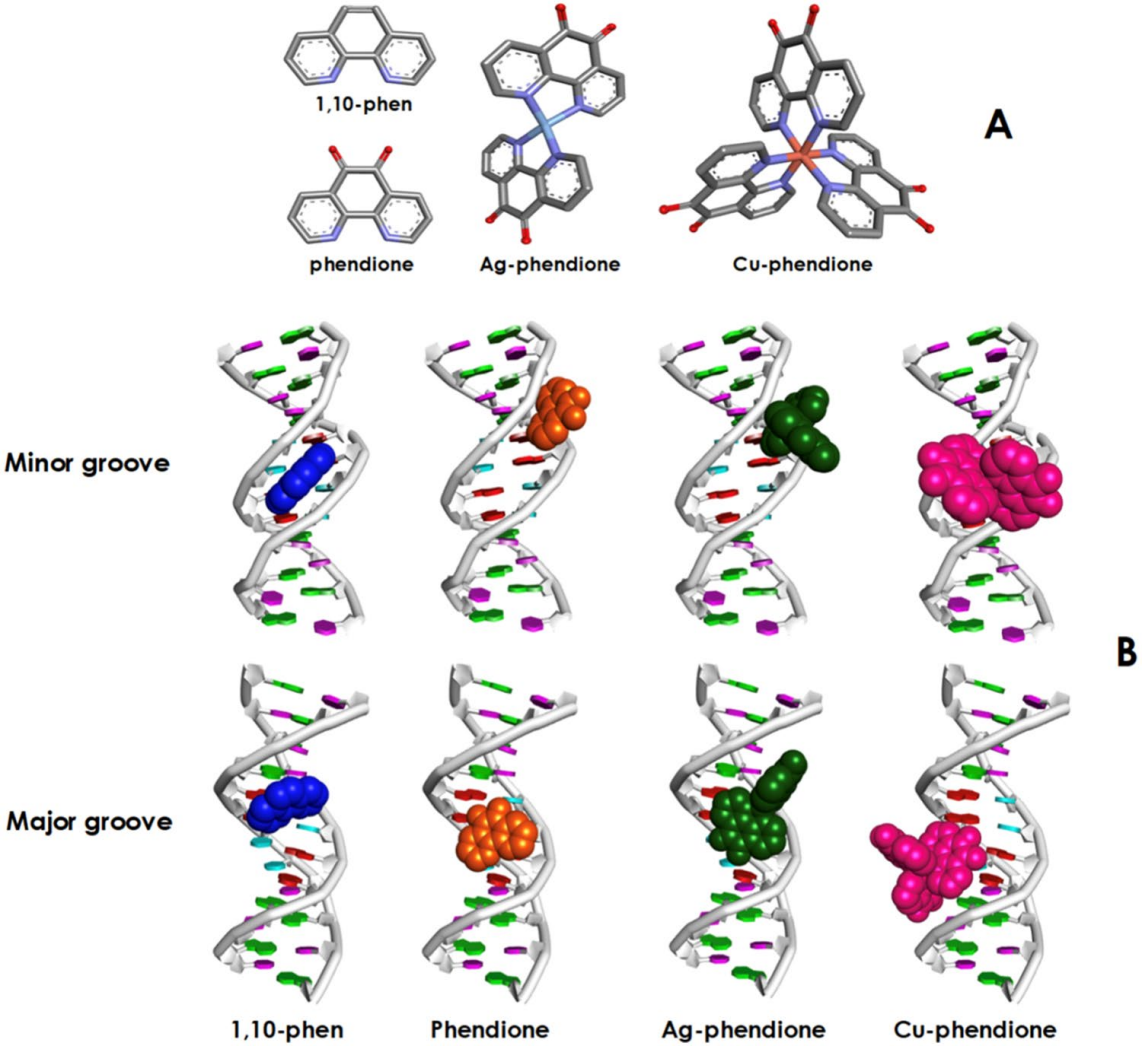
Molecular structures of 1,10-phenanthroline (1,10-phen), 1,10-phenanthroline-5,6-quinone (phendione) along with copper(II) and silver(I) phendione (**A**). Major and minor grooves of DNA molecules and the best docking poses for DNA with the test compounds (**B**)

1,10-Phen was obtained from Sigma-Aldrich (USA), and phendione, [Ag(phendione)_2_]ClO_4_ (Ag-phendione) and [Cu(phendione)_3_](ClO_4_)_2_.4H_2_O (Cu-phendione) (Fig. 1A) were prepared as previously reported [28, 29].

### Molecular docking

Molecular docking analysis of DNA with 1,10-phen, phendione, Ag-phendione and Cu-phendione were calculated using AutoDock Vina 1.1.2 program [30]. The 3D atomic coordinates of the test compounds were computed by Discovery Studio, v20 (Accelrys, USA) and their rigid root was generated using AutoDockTools (ADT) [31], setting all possible rotatable bonds defined as active by torsions. In addition, ADT was used to prepare the receptor (DNA–PDB: 1bna) input file by merging non-polar hydrogen atoms, adding partial charges and atom types. The grid center at the center of mass (*x*-, *y*-, and *z*-axes) of DNA major and minor groove was 21.416 Å × 19.370 Å × 8.812 Å and 6.936 Å × 19.732 Å × 11.931 Å, respectively. The grid dimension used for DNA major groove was 18 Å × 38 Å × 24 Å and minor groove was 16 Å × 34 Å × 32 Å. The binding model was searched out from 10 different conformers for each ligand.

### Electrospray ionization mass spectrometry (ESI–MS) analyses

ESI–MS spectra were recorded using a Thermo Fisher Exactive Orbitrap mass spectrometer coupled to an Advion TriVersa Nanomate injection system with samples prepared as described below. Accurate mass spectrometry was conducted on a MaXis HD quadrupole electrospray time-of-flight (ESI-QTOF) mass spectrometer (Bruker Daltonik GmbH, Bremen, Germany), using a glass syringe (Hamilton) and syringe pump (KD Scientific, Model 781100) for infusions at a flow rate of 3 μL/min. Analyses were performed in ESI positive mode with the capillary voltage was set to 4500 V, nebulizing gas at 0.6 bar, drying gas at 4 L/min at 180 °C in each case. The TOF scan range was from 75 to 1600 mass-to-charge ratios (*m/z*). The MS instrument was calibrated using an infusion of sodium formate calibrate solution. The calibrant solution consisted of 3 parts of 1 M NaOH to 97 parts of 50:50 water:isopropanol with 2% formic acid. Data processing was performed using the Compass Data Analysis software version 4.3 (Bruker Daltonik GmbH, Bremen, Germany).

To study the stability of Cu(II) complexes, ESI–MS studies were performed in situ in the absence of reductant over 72 h. Accurate mass spectrometry analyses was carried out using the method described by McStay et al. [32]. Briefly, in a total volume of 1 mL, stock solutions of 1,10-phen or phendione (4 mM) in THF:H_2_O, 50:50 were mixed with copper(II) nitrate trihydrate (1.3 mM) in ratio 3:1. Samples were incubated at 37 °C for 30 min and further dilution was made as required to perform ESI–MS analysis (to reach a final concentration of 1 mg/mL). ESI–MS spectra were recorded at time points: 0 h, 24 h, 48 h, and 72 h.

To study the solution stability of both copper complexes during redox processes, a second experiment was designed to monitor both complexes in situ in the presence of reductant Na-*L*-ascorbate (Na-*L*-asc). Both complexes were prepared as described above and spectra were recorded before and after reduction with 3 mM Na-*L*-asc (t = 0 h). After 24 h, a second titration of 3 mM Na-*L*-asc was performed and the spectra for each solution recorded. At 48 h, ESI–MS spectra were recorded before and after a third addition of 3 mM of Na-*L*-asc. A final measurement was then taken at 72 h.

### DNA-binding studies

#### Competitive ethidium bromide (EtBr) displacement

To analyze the binding affinity between DNA and test compounds, the competitive EtBr (Sigma-Aldrich) displacement was employed as previously reported [33]. Briefly, a solution of 20 µM calf thymus DNA (ctDNA, Invitrogen 15633-019, Ɛ_260_ = 12.824 M (bp)^−1^/cm) and 25.2 µM EtBr was prepared in 80 mM HEPES buffer and 40 mM NaCl, pH 7.2. The test compounds were prepared in DMSO at 4 mM. Assays were carried out in 96-well microplates (Corning, USA) and the fluorescence readings were recorded (Ex: 530 nm, Em: 590 nm; Bio-Tek Synergy HT Multi-mode). Triplicate titrations were performed, and the apparent binding constants were calculated using *K*_app_ = *K*_e_ × 12.6/*C*_50_, where, *K*_e_ = 9.5 × 10^6^ M^−1^ and *C*_50_ is the concentration of test compounds required to reduce the EtBr fluorescence by half [33].

#### Fluorescence quenching of EtBr-DNA and Hoechst 33258-DNA

Experiments were conducted in accordance to the method reported by Molphy et al. [28]. Fluorescence readings were recorded using a Bio-Tek Synergy HT Multi-mode microplate reader at an excitation wavelength of 530 or 360 nm and an emission wavelength of 590 or 460 nm for EtBr and Hoechst fluorescence detection, respectively. Repeated aliquots were added until the fluorescence was 30–40% of the initial control. Each drug concentration was measured in triplicate, on at least two independent experiments. From a plot of fluorescence versus added drug concentration, the *Q* value is given by the concentration required to effect 50% removal of the initial fluorescence of the bound dye [28].

### Topoisomerase I inhibition assay

pUC19 plasmid DNA (400 ng; NEB, N3041) was exposed to increasing concentrations of each complex (2.5–400 µM) for 30 min at 20 °C in a final volume of 20 µL containing 80 mM HEPES buffer, 10 × CutSmart® buffer, and 100 × BSA (NEB). One unit of topoisomerase I (*E. coli*) (NEB) was added to the mixture and incubated for 20 min at 37 °C. The reaction was stopped through the addition of 0.25% SDS and 250 µg/mL protein kinase and further incubated for 30 min at 50 °C. The 6 × loading dye was added and topoisomers of DNA were separated by electrophoresis in 1 × TBE buffer for 180 min/40 V and 150 min/50 V. The agarose gel (1.2%) was post-stained using an EtBr bath and photographed using a SynGene G:BOX mini6 [34].

### DNA damage studies

#### DNA cleavage in the absence of reductant

pUC19 (400 ng) was exposed to 10–75 µM of each complex in a final volume of 20 µL containing 80 mM HEPES buffer and 25 mM NaCl. Reactions were incubated at 37 °C in darkness for either 3 h (Cu-phendione) or 24 h (Ag-phendione). The 6 × loading dye was added to each sample prior to loading on a 1% agarose gel containing 4 µL SYBR Safe. Electrophoresis was carried out at 70 V for 60 min in 1 × TAE buffer and photographed.

#### DNA cleavage in the presence of reductant

pUC19 (400 ng) was exposed to varying concentrations (5–50 µM) of Cu-phendione in the presence of 25 mM NaCl and 1 mM Na-*L*-asc and incubated at 37 °C for either 30 or 60 min. Electrophoresis was carried out at 70 V for 60 min in 1 × TAE buffer and photographed.

#### Kinetic DNA cleavage study

pUC19 (400 ng) was exposed to either 30 or 40 µM of Cu-phendione in the presence of 25 mM NaCl and 1 mM Na-*L*-asc. Incubation times varied from 10 to 60 min and were performed at 37 °C in darkness. Electrophoresis was conducted at 70 V for 60 min in 1 × TAE buffer and photographed. To quantify DNA damage using band densitometry, 40 µM of Cu-phendione was exposed to DNA in the presence of Na-*L*-asc (10–60 min). This experiment was conducted in triplicate and band densitometry was analyzed on the SynGene G:BOX mini6 using SynGene Gene Tools software.

#### DNA cleavage in the presence of reactive oxygen species (ROS) scavengers

pUC19 (400 ng) was treated with increasing complex concentrations in the presence of 25 mM NaCl, 1 mM Na-*L*-asc and a range of ROS scavengers: 4,5-dihydroxy-1,3-benzenedisulfonic acid (Tiron, O_2_^•−^, 10 mM), KI (H_2_O_2_, 10 mM), DMSO (^•^OH, 10%) and D_2_O (^1^O_2_, 10%) in 80 mM HEPES. Reactions were incubated at 37 °C for 60 min in darkness.

### Displacement of propidium iodide (PI) from the bacterial genomic DNA

*Pseudomonas aeruginosa* (ATCC 27853) cells were cultured in LB broth at 37 °C for 24 h. Then, 10^6^ colony-forming units (CFUs)/mL in saline solution (0.85% NaCl) were heated at 72 °C for 30 min to inactivate the bacteria and allow for passive internalization of PI. The permeabilized bacteria were incubated with 20 mM PI for 1 h in the dark followed by three washes with saline. Subsequently, the cells were incubated with each compound (50, 250, 500 and 1000 mM) for 1 h. Finally, the fluorescence reading from each sample was analyzed in a flow cytometer (FACS Calibur, BD Bioscience, USA) equipped with a 15-mW argon laser emitting at 488 nm. The reduction of fluorescence of the treated systems compared to the untreated control reflects the displacement of the PI bound to the bacterial DNA by the compounds [35].

### Bacterial genomic DNA fragmentation

First, bacteria (10^6^ CFUs/ml) were grown in LB broth in the absence and in the presence of 2 × MIC value (15 μM) [11] of Cu-phendione [9] or H_2_O_2_ (17.5 mM) for 5 h at 37 °C under shaking (150 rpm). Bacteria were then harvested by centrifugation (4000 × *g*/10 min/4 °C) and washed with saline (3 ×). To evaluate bacterial genomic DNA integrity by electrophoretic mobility, the genomic DNA was extracted using the Gentra Puregene Yeast and Bacteria Kit (Qiagen, USA) according to the manufacture instructions. DNA samples were subjected to electrophoresis (1% agarose gel in TBE buffer) for 90 min at 100 V and then stained using an EtBr bath and photographed.

### TUNEL assay

Bacteria grown in LB broth as reported above were fixed with 4% paraformaldehyde for 30 min. DNA fragmentation was evaluated using the DeadEndTM Fluorometric TUNEL System Kit (Promega, USA) following the manufacturer's recommendations and then analyzed by flow cytometry. The data obtained were analyzed using Flowing software 2.5.1.

### Statistics

The results were evaluated by analysis of variance (ANOVA) and Dunnett’s multiple comparison tests using GraphPad Prism 8 computer software (GraphPad Software Inc., USA). In all analyses, *p* values ≤ 0.05 were considered statistically significant.

## Results and discussion

### Copper- and silver-phendione complexes interact with double-stranded DNA: in silico experiments

Molecular docking analysis was performed to identify interactions and binding affinities of 1,10-phen, phendione, Ag-phendione and Cu-phendione with both major and minor grooves of double-stranded DNA. The best docking poses for DNA with each compound were visualized (Fig. 1B). Details of the best binding simulations and docking affinities, nucleic acid interactions, type of interaction and geometry distance of each ligand and double-stranded DNA were displayed in the Supporting Material (Tables S1 and S2). The results showed that test compounds were able to bind to the DNA by means of hydrogen bonds and hydrophobic interactions. In addition, Cu-phendione displayed the ability to promote electrostatic interactions with DNA molecules. Conversely, the interactions of DNA minor grooves with 1,10-phen and phendione were based on hydrogen bonds. Ag-phendione and Cu-phendione showed particular behaviors, exhibiting additional ability to establish hydrogen bonds and electrostatic interactions with the minor groove. Hydrophobic interactions were only observed between Cu-phendione and DNA molecules. Kamran et al. [36] have applied computational methods to investigate the interaction of DNA and binuclear Cu(II) complexes represented by the general formula {(DMSO)Cu(µ-L)_4_Cu(DMSO)} and {(1,10-phen)(L)Cu(µ-L)_2_Cu(L)(1,10-phen)}, where *L* = 2-bromophenyl acetate. It was observed that both binuclear Cu(II) complexes formed H–π interaction with adenine and guanine residues of the DNA [36].

### Copper- and silver-phendione complexes interact with double-stranded DNA: in vitro analysis

**Fig. 2:**
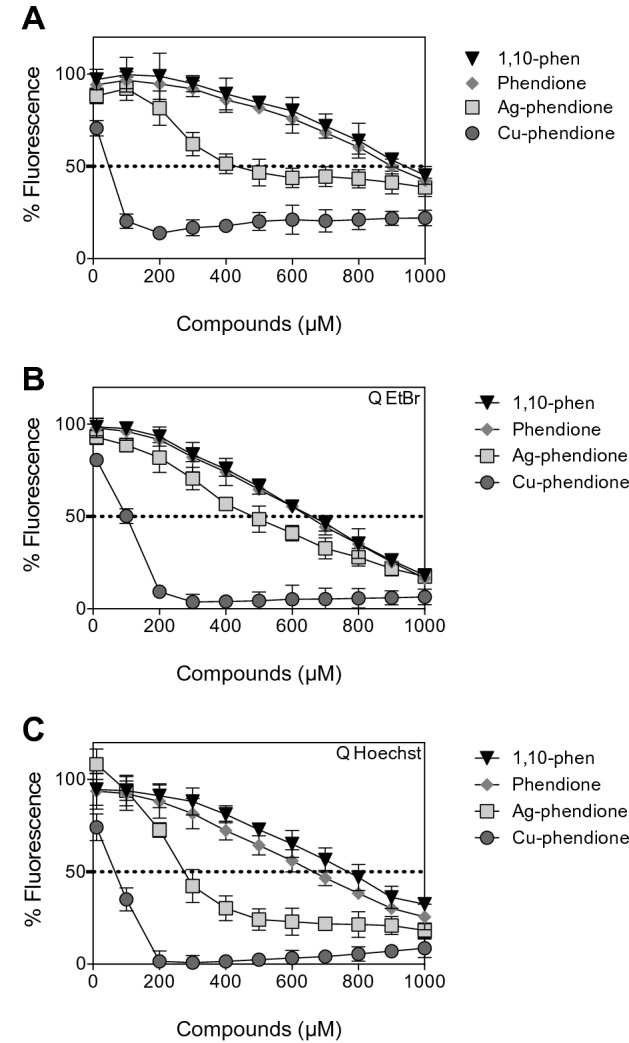
Interaction between 1,10-phen and phendione-based compounds and calf thymus DNA (ctDNA). Competitive EtBr displacement assays (**A**), fluorescence quenching of EtBr (**B**), and Hoechst 33258 (**C**) with ctDNA. Fluorescence readings were recorded in a microplate reader and expressed as the percentage of fluorescence in comparison to each control, which was read in the absence of the test compounds. The dashed lines represent 50% fluorescence of control. Data points are presented as an average of triplicate measurements ± SD

DNA-binding constants of the Ag-phendione and Cu-phendione were determined indirectly by high throughput saturation binding analysis (Fig. 2, Table 1) that employs the heterocyclic EtBr as a reporter molecule. The method involved treating calf thymus DNA (ctDNA; 20 µM) in 80 mM HEPES buffer (pH 7.2) containing 40 mM NaCl with a saturated concentration of EtBr (25.2 µM) prior to the titration of tested complex. Both Ag-phendione and Cu-phendione were found to bind ctDNA with *K*_app_ values of ca. 2.8 × 10^5^ and 2.6 × 10^6^ M^−1^, respectively (note: *K*_app_ = *K*_e_ × 12.6/*C*_50_, where *K*_e_ = 9.5 × 10^6^ M^−1^ and *C*_50_ is the concentration of test compounds required to reduce the EtBr fluorescence by half). The binding constants are in line with a number of Cu(II)-phenanthroline systems previously reported [36] and it should also be noted that the influence of charge may play a role in the higher binding affinity associated with the Cu-phendione complex, which carries a 2+ cationic charge. Furthermore, the binding constant of Cu-phendione is similar to that identified for the minor groove binding agent netropsin tested under similar conditions but is an order of magnitude lower that actinomycin D (Table 1) [33]. Competitive fluorescence quenching experiments in the presence of limited bound Hoechst 33258 (minor groove binder) or EtBr were carried out to identify a preference for DNA-binding sites. This experiment revealed a preference for both complexes to bind ctDNA at the minor groove as quenching (*Q*) values obtained in the presence of Hoechst 33258 were over half that of concentrations required to quench fluorescence in the presence of EtBr. A similar effect was reported in a series of bis-chelate Cu^2+^-phenanthroline–phenazine cationic complexes, where the systematic extension of the ligated phenazine ligand was found to influence DNA recognition [28]. In accordance, it was demonstrated that the copper 1,10-phen-derivative has a higher binding affinity to DNA than the 1,10-phen itself [38]. That study reported 1,10-phen (up to 200 μM) had little effect on ctDNA pre-exposed to EtBr fluorogenic dye [38]. However, the addition of ([Cu(1,10-phen)_x_]^2+^) to DNA system reduced the *Q*_EtBr_ to 2.7 μM, suggesting that the interaction between 1,10-phen and DNA is dependent on the presence of a metal ion. Furthermore, Cu-phendione exhibited the lowest concentration that inhibits 50% fluorescence (*Q*_EtBr_ = 100.7 μM and *Q*_Hoechst_ = 66.0 μM), which suggests that this compound has a dual-mode of interaction with DNA, being able to intercalate into the compact array of stacked bases as well as partially groove bind to the DNA. Previously, it was reported that [Ag(PDT)_2_]ClO_4_.2MeOH and [Cu(PDT)_2_](ClO_4_)_2_ displayed high DNA-binding affinities (*K*_app_ = 7.60 × 10^6^ M^−1^ and 7.62 × 10^6^ M^−1^, respectively), and similarly to Cu-phendione, [Ag(PDT)_2_]ClO_4_.2MeOH and [Cu(PDT)_2_](ClO_4_)_2_ were able to act as intercalators and as a minor groove binders (respectively, *Q*_EtBr_ = 18.2 μM and 18.6 μM, *Q*_Hoechst_ = 24.7 μM and 18.0 μM) [33]. Likewise, Kellett et al. [39] reported that both [Cu(ph)(1,10-phen)]0.2H_2_O and [Cu(ph)(2,2’-bipy)]0.2H_2_O (where ph = *o*-phthalate) bind to duplex DNA as either a semi-intercalating agent or by binding to the minor groove (*K*_app_ = 1.2 × 10^5^ M^−1^ and 1.1 × 10^5^ M^−1^, respectively).

**Table 1:** DNA-binding properties

Compounds	*C*_50_ (μM)^a^	*K*_app_^b^	*Q* EtBr^c^	*Q* Hoechst (µM)^c^
Actinomycin D [28]	4.1	2.92 × 10^7^	4.8	26.3
Netropsin [28]	46.27	2.50 × 10^6^	20.0	2.4
1,10-phen	941.0	1.27 × 10^5^	659.9	768.6
Phendione	899.1	1.33 × 10^5^	648.8	664.0
Ag-phendione	429.0	2.79 × 10^5^	482.2	274.4
Cu-phendione	46.9	2.55 × 10^6^	100.7	66.0

Apparent ctDNA binding constants (*K*_app_) determined using competitive ethidium bromide (EtBr) quenching and fluorescence quenching (Q) of DNA bound with either EtBr or Hoechst 33258. Classical DNA-binding drugs of actinomycin D and netropsin tested under identical conditions [28] are provided for reference

^a^*C*_50_ = concentration required to reduce fluorescence by 50%,

^b^*K*_app_ = *K*_e_ × 12.6/*C*_50_ where *K*_e_ = 9.5 × 10^6^ M (bp)^−1^,

^c^*Q* = displacement of 50% initial fluorescence from DNA-bound dye

### Cu-phendione induces topoisomerase I-mediated DNA relaxation

**Fig. 3:**
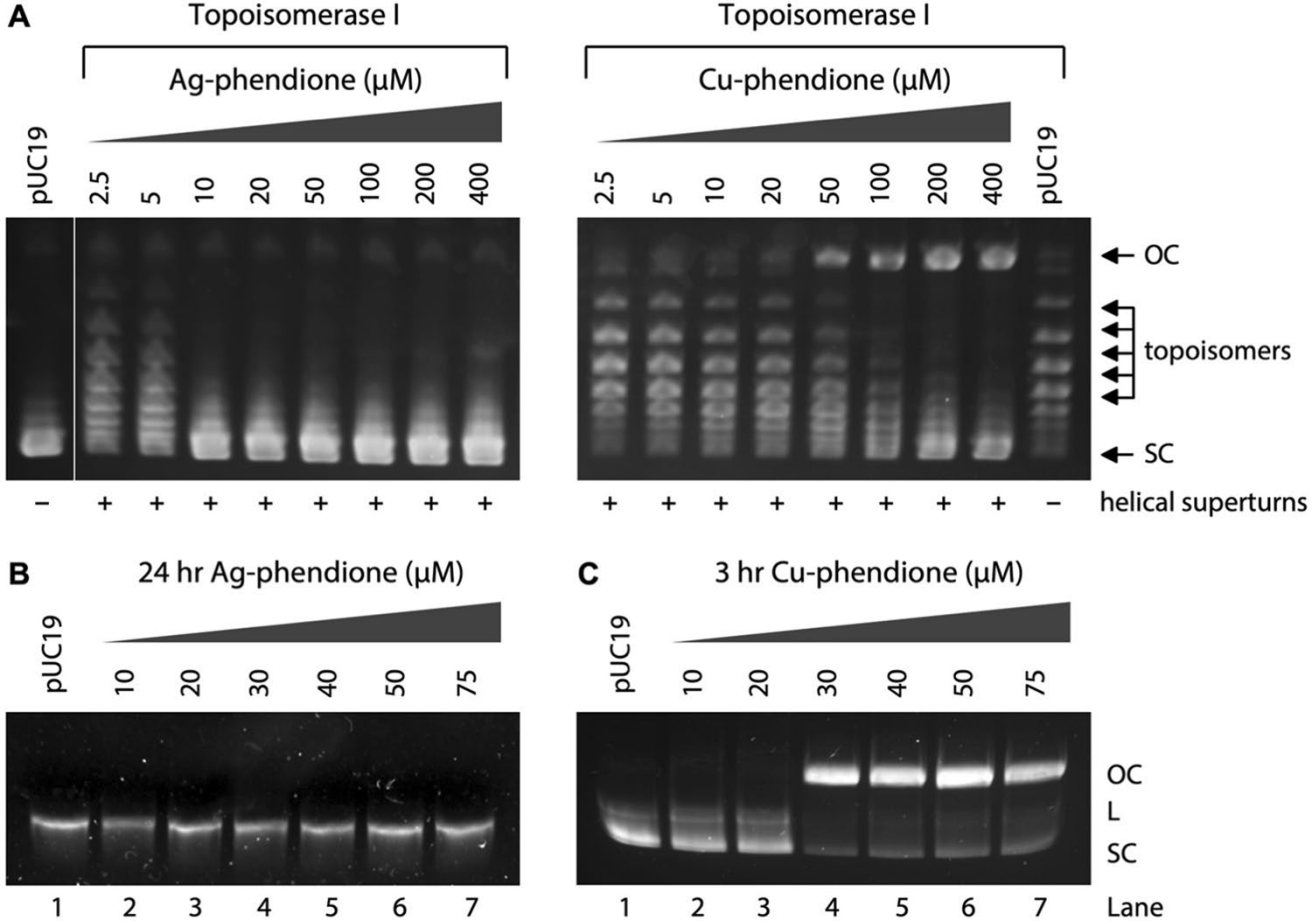
Release of topological tension from supercoiled pUC19 using the topoisomerase I-mediated relaxation assay in the presence Ag-phendione (**A**) or Cu-phendione (**B**). pUC19 treated with increasing concentrations of Ag-phendione in the absence of reductant over 24 h (**C**). pUC19 treated with increasing concentrations of Cu-phendione in the absence of reductant over 3 h (**D**)

To characterize the intercalative activity of the Ag-phendione and Cu-phendione, the topoisomerase I-mediated DNA relaxation assay was performed on supercoiled (SC) plasmid DNA (Fig. 3A). Plasmid unwinding by both complexes was examined between 0.5 and 400 µM. Cu-phendione was found to first unwind negatively SC DNA prior to introducing DNA damage at concentrations greater than 50 µM. This effect has also been observed in mono-nuclear systems including Cu-TPMA-N,N’ (where N,N’ = 1,10-phen, DPQ and DPPZ) and in *di*-nuclear Cu(II) systems such as Cu-Oda and Cu-Terph [40, 41]. In the presence of increasing concentrations of Ag-phendione, pUC19 became wound in the opposite direction with positive supercoils observed at 10 µM. This electrophoretic mobility shift assay revealed Ag-phendione treated DNA remained intact up to the maximum exposure concentration (400 µM).

### Cu-phendione promotes oxidative DNA damage

**Fig. 4:**
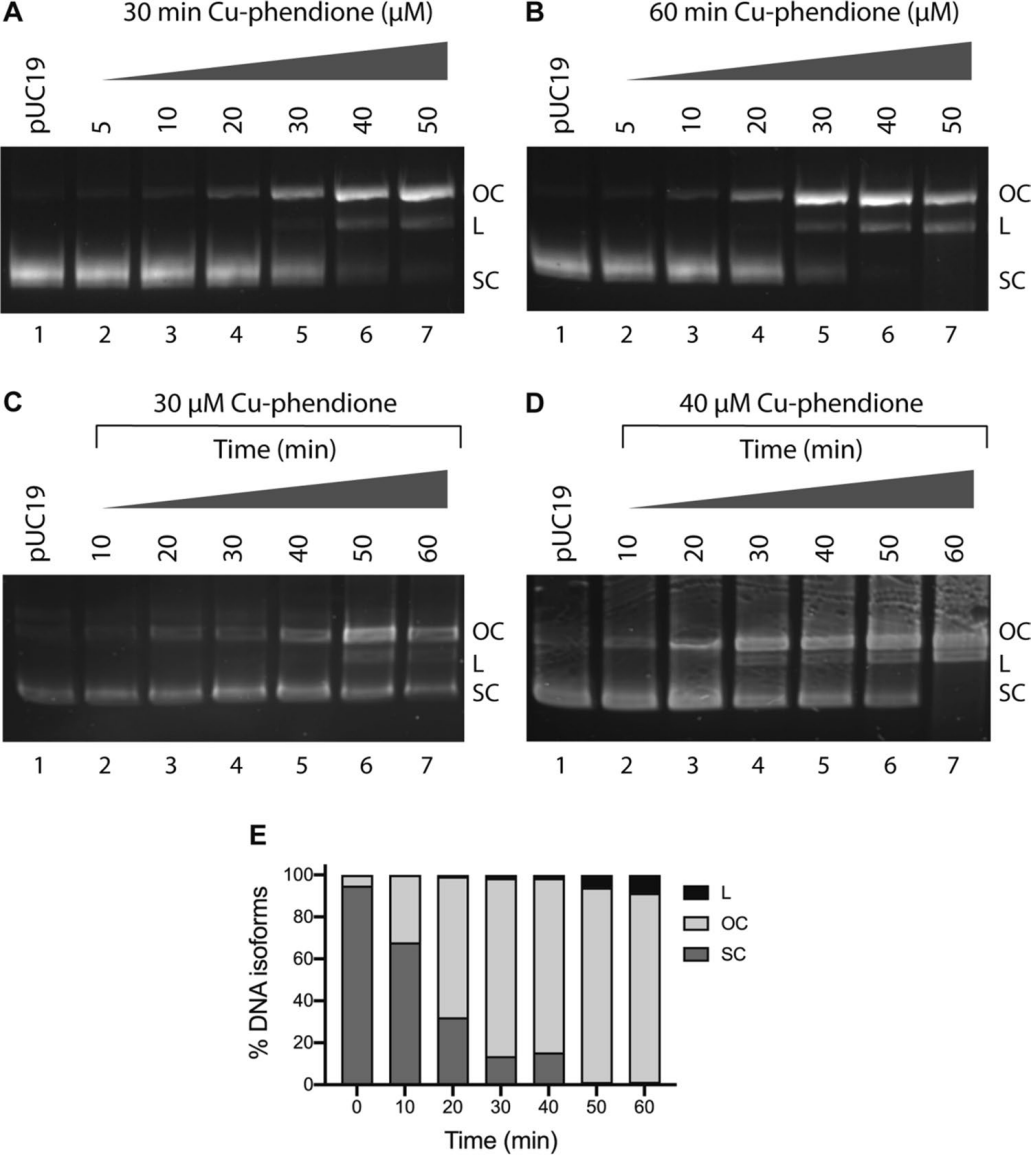
pUC19 DNA treated with increasing concentrations of Cu-phendione for 30 min (**A**) and 60 min (**B**) in the presence of 1 mM Na-*L*-ascorbate. Kinetic DNA damage study over 60 min in the presence of reductant at 30 µM (**C**) and 40 µM (**D**) Cu-phendione exposure. DNA densitometry analysis of pUC19 treated with 40 µM Cu-phendione over 60 min (**E**)

A number of experimental conditions were explored during DNA damage investigations of both metal-phendione complexes including the (i) presence/absence of exogenous reductants, (ii) complex exposure concentration/duration and (iii) influence of scavenging species. First, the DNA damage profiles of both complexes were assessed in the absence of reductant. Over a 24-h exposure period, Ag-phendione failed to induce damage up to 75 µM and it was, therefore, not further investigated (Fig. 3B). As anticipated, Cu-phendione was more active and found to cleave SC plasmid to OC at 30 µM in a shorter time frame (3 h) in the absence of reductant (Fig. 3C). In the presence of exogenous reductant (1 mM Na-*L*-asc), the active Cu(I) species catalyzes the production of ROS at the DNA interface resulting in enhanced oxidative chemical nuclease activity. A 30-min incubation carried out in the range of 5–50 µM Cu-phendione resulted in the stepwise conversion of SC DNA to both OC and L forms, with three isoforms becoming visible at 40 µM treatment (Fig. 4A, lane 6). A follow-up experiment was conducted where the time frame was extended out to 60 min and it was again possible to detect SC, OC and L forms, but at a lower complex concentration of 30 µM (Fig. 4B, lane 5). In an effort to improve separation of DNA isoforms, a kinetic experiment was preformed, where 400 ng of plasmid was treated with 30 µM Cu-phendione with measurements taken every 10 min for a total of 60 min; however, no significant enhancement in separation was observed (Fig. 4C). The concentration in the kinetic experiment was increased to 40 µM with three isoforms of pUC19 plasmid DNA qualitatively observed by electrophoresis (Fig. 4D). The experiment was repeated in triplicate (Fig. S1) and isoforms were quantitatively determined by band densitometry analysis (Fig. 4E). Traces of all three DNA isoforms were detected between 20 and 40 min complex exposures. At 50 min of Cu-phendione exposure, SC DNA was fully depleted and plasmid DNA was fully converted to OC and L forms.

**Fig. 5:**
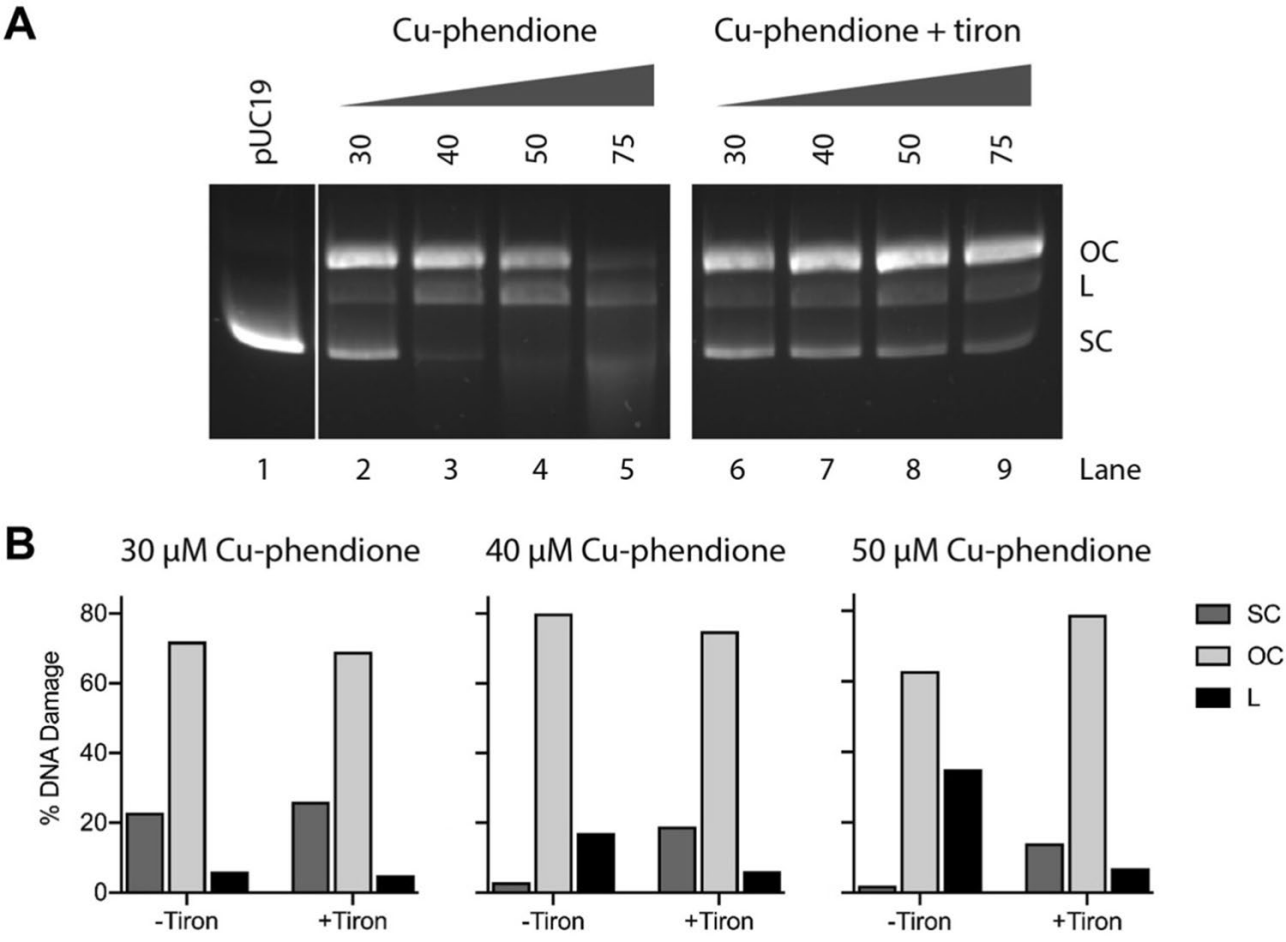
pUC19 DNA treated with increasing concentrations of Cu-phendione in the presence of 1 mM Na-*L*-ascorbate (lanes 2–5) and 10 mM of scavenging species Tiron (lanes 6–9) (**A**). Representative band densitometry analysis of DNA isoforms in the absence and in the presence of Tiron (**B**)

To shed further light on the ROS species involved in DNA damage by Cu-phendione, oxidative DNA cleavage was triggered in the presence of a variety of ROS specific scavengers and stabilizers including tiron (O_2_^•−^), *D*-mannitol (^•^OH), KI (H_2_O_2_) and D_2_O (^1^O_2_ stabilizer). A preliminary study indicated that ^•^OH, H_2_O_2_ and ^1^O_2_ play only a minor role in oxidative mechanism of Cu-phendione and they were not further investigated (data not shown). However, when the O_2_^•−^ radical was scavenged by tiron, DNA damage was significantly impeded. Sequestering the superoxide radical with tiron resulted in significant protection of plasmid DNA. A delayed onset of OC-DNA formation and protection of SC DNA were particularly evident. It was also possible to visualize all three isoforms (SC, OC and L) up to 75 µM of complex exposure (Fig. 5, lanes 6–9). Sequestering the O_2_^•−^ radical has been recently found to have a significant impact on DNA damage induced by mono-nuclear Cu-TPMA-phenanthrene and Cu-DPA-phenanthrene systems [39]. Similarly, the nuclease activity of Cu-phen-CipA (CipA = ciprofloxacin) was inhibited by KI (H_2_O_2_ scavenger), NaN_3_ (^1^O_2_ scavenger), DMSO (^•^OH scavenger) and Tiron (O_2_^•−^ scavenger) [42]. Oxidative mechanisms were crucial for the artificial metallo-nuclease activity of [Cu(DPQ)_2_(NO_3_)](NO_3_) (DPQ = dipyrido[3,2-f:2′,3′-h]quinoxaline) [43].

### Solution stability of Cu-phen and Cu-phendione complexes

**Fig. 6:**
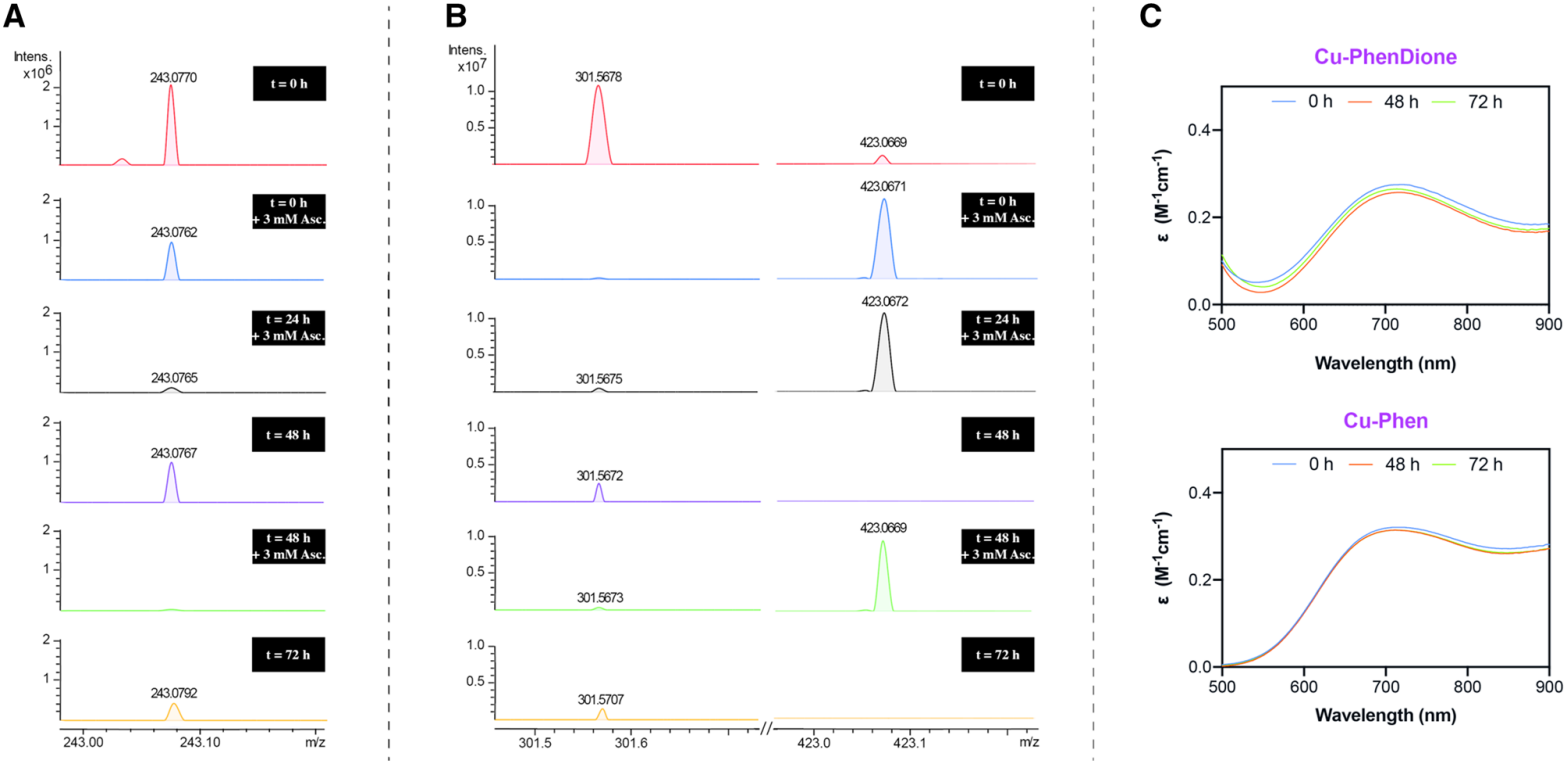
In situ ESI–MS analyses of 3:1 Cu(II):phendione after reduction with 3, 6, and 9 mM of Na-*L*-ascorbate over 72 h (**A**). In situ ESI–MS analyses of 3:1 Cu(II):phen after reduction with 3, 6, and 9 mM Na-*L*-ascorbate over 72 h (**B**). In situ UV–Vis stability study of 5 mM solutions of 3:1 Cu(II) nitrate:phen and Cu(II) nitrate:phendione recorded in CH_3_CN:H_2_O (50:50) over 72 h (**C**)

To identify reasons for the chemical nuclease activity of Cu-phendione, detailed ESI–MS and UV–Vis absorbance measurements were undertaken and compared with Cu-phen. Here, 1,10-phen or phendione (4 mM) were dissolved in THF:H_2_O (50:50) and incubated with copper(II) nitrate trihydrate (1.3 mM) in a 3:1 molar ratio at 37 °C for 30 min. Samples of each spectra were then recorded every 24 h over a 72 h period where predominant species of [Cu(phendione)_2_]^2+^ and [Cu(phen)_3_]^2+^ were characterized and remained stable over the time-course measurements (Figs. S2 and S3). This experiment was then performed using UV–Vis spectroscopy and no significant change to the *d*–*d* absorbance properties of either complex solution was observed over time (Fig. 6C). A second in situ ESI–MS experiment was then undertaken where spectra of both 3:1 solutions were recorded after reduction with 3, 6, and 9 mM of Na-*L*-asc introduced over 72 h (Fig. 6A, B). After addition of 3 mM of ascorbate (*t* = 0 h) the [Cu(phendione)_2_]^2+^ cation ([M + 2H]^+^  = 243.07 m/*z*) diminishes (but is still detectable) while the [Cu(phen)_3_]^2+^ cation ([M]^+^  = 301.56 m/*z*) is consumed with concomitant generation of [Cu(phen)_2_]^+^ ([M]^+^  = 423.06 m/*z*). The addition of a second aliquot of 3 mM of ascorbate at 24 h ablates the [Cu(phendione)_2_]^2+^ cation with no further changes to the Cu-phen solution observed. Interestingly, after 48 h, the [Cu(phendione)_2_]^2+^ cation begins to re-emerge (Fig. 6A), while by comparison, only a small fraction of the [Cu(phen)_3_]^2+^ and none of the [Cu(phen)_2_]^+^ cation was detectable (Fig. 6B). The addition of a further aliquot of ascorbate (3 mM) at 48 h then ablated [Cu(phendione)_2_]^2+^ and, in the phen solution, leads to the formation of [Cu(phen)_2_]^+^. The final measurement at 72 h revealed partial re-emergence of [Cu(phendione)_2_]^2+^ (Fig. 6A) where, in parallel, a smaller fraction of [Cu(phen)_3_]^2+^ and no detectable [Cu(phen)_2_]^+^ was found (Fig. 6B). Results here suggest that although both complexes form stable in situ species—namely Cu(II) *bis*-phendione and Cu(II) *tris*-phen complexes—differences emerge in the presence of a reductant. First, [Cu(phendione)_2_]^2+^ appears to have greater solution stability and is less easily reduced compared to [Cu(phen)_3_]^2+^. This phendione complex also begins to re-emerge as the solution becomes oxidised over 48 h and 72 h periods. In contrast, very little of the initial [Cu(phen)_3_]^2+^ complex regenerates and, after prolonged incubation of 48 and 72 h, neither Cu(II) or Cu(I) phen complexes are detectable. Although these conditions reveal the [Cu(phendione)_2_]^2+^ complex is potentially more stable than [Cu(phen)_3_]^2+^, care must be taken in interpreting these in situ data in the absence of DNA.

### Cu-phendione interacts with pseudomonal DNA and promotes oxidative damage

Having observed the in silico*/*in vitro interactions between Cu-phendione and DNA molecules, the direct harmful action of this complex on pseudomonal DNA was investigated, since Cu-phendione had a powerful anti-*P. aeruginosa* action as previously reported by our group [9]. Initially, *P. aeruginosa* cells were heat-inactivated to permeabilize them without disrupting bacterial architecture, followed by sequential incubation with the DNA intercalator PI and different concentrations of test compounds; finally, the fluorescent cells were analyzed by flow cytometry. Our results revealed that 1,10-phen and phendione were not able to significantly displace the PI dye from DNA, indicating the weak or lack of interaction between these compounds and bacterial DNA (Table 2). Contrarily, Ag-phendione and Cu-phendione avidly displaced PI bound to pseudomonal DNA in a typically dose-dependent manner; both complexes at 1000 μM, for example, significantly reduced the cell-associated fluorescence around 51% and 62%, respectively (Table 2).

**Table 2:** Interaction between phendione-based compounds and pseudomonal DNA

Systems	Compounds (µM)	Mean fluorescence intensity	% Fluorescent cells
Bacterial cells	–	8.55 ± 0.07	0.05 ± 0.07
Bacterial cells + PI	–	32.85 ± 0.07	63.05 ± 0.07
Bacterial cells + 1,10-Phen	1000	8.2 ± 0.07	0.1 ± 0.07
Bacterial cells + phendione	1000	8.1 ± 0.06	0.1 ± 0.07
Bacterial cells + Ag-phendione	1000	8.7 ± 0.07	0.1 ± 0.07
Bacterial cells + Cu-phendione	1000	9.0 ± 0.08	0.1 ± 0.07
Bacterial cells + PI + 1,10-Phen	1000	35.35 ± 4.31	64.65 ± 2.47
	500	35.95 ± 0.78	65.10 ± 1.41
	250	33.70 ± 3.11	63.95 ± 2.47
	50	24.55 ± 7.14	54.10 ± 9.19
Bacterial cells + PI + phendione	1000	28.55 ± 1.48	60.45 ± 0.35
	500	30.30 ± 2.89	61.40 ± 2.69
	250	27.80 ± 1.41	59.60 ± 1.56
	50	32.90 ± 3.68	63.51 ± 2.69
Bacterial cells + PI + Ag-phendione	1000	15.25 ± 0.07*	34.40 ± 0.28*
	500	16.25 ± 0.50*	39.05 ± 0.64*
	250	20.55 ± 1.06*	43.50 ± 3.54*
	50	21.65 ± 2.90*	47.85 ± 6.01*
Bacterial cells + PI + Cu-phendione	1000	12.65 ± 0.07*	24.35 ± 0.35*
	500	13.05 ± 0.07*	29.90 ± 0.14*
	250	16.80 ± 2.40*	40.85 ± 4.45*
	50	21.85 ± 0.07*	51.30 ± 0.85*

*Significant difference of the treated systems compared to the control (*P* < *0.05*—analysis of variance one-way (ANOVA) (Dunnett’s multiple comparison test)

**Fig. 7:**
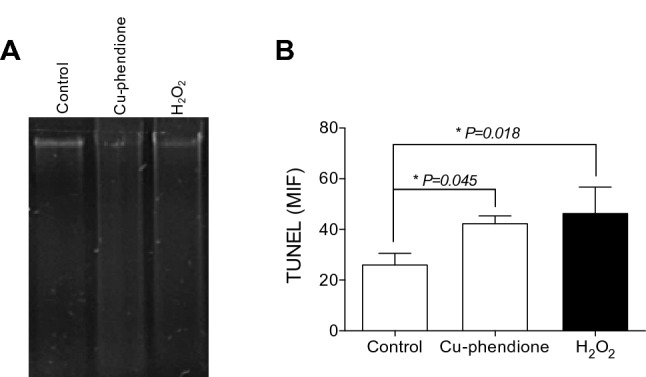
Cu-phendione induces oxidative DNA damage in *P. aeruginosa.* The fragmentation of pseudomonal DNA was evaluated by the electrophoretic profile of genomic DNA (control) obtained from ATCC 27853 cells cultured with either 2 × MIC of Cu-phendione or 17 mM H_2_O_2_ (**A**). Bacterial cells exposed to Cu-phendione (2 × MIC; 15 µM) or 17 mM H_2_O_2_ were labeled with the TUNEL probe for DNA detection with the 3'-OH end; DNA fragmentation was evaluated by flow cytometry and expressed as mean fluorescence intensity (MFI) (**B**). Data points are displayed as an average of triplicate measurement. The asterisks (∗ *P* < 0.05, one-way ANOVA, Dunnett’s multiple comparison test) denote the statistically significant difference among Cu-phendione-treated and H_2_O_2_-treated systems and the untreated one

Subsequently, the DNA fragmentation profile was verified by agarose gel using genomic DNA extracted from *P. aeruginosa* cultures treated with bactericidal concentrations of Cu-phendione [9]. After 5 h of treatment, the bactericidal concentration of Cu-phendione (15 μM) and H_2_O_2_ (17 mM) induced the fragmentation of *P. aeruginosa* genomic DNA, as visualized in the agarose gel as a smear corresponding to degradation of intact DNA molecules in small molecular weight fragments (Fig. 7A). In parallel, Cu-phendione-mediated DNA fragmentation was confirmed by TUNEL assay. This method relies on the attachment of modified nucleotides (FITC-labeled) into the 3'-hydroxyl terminal of DNA double-strand breaks [44]. Similarly, both Cu-phendione and H_2_O_2_ showed, respectively, an increase of 62.8% and 78.5% in the incorporation of fluorescent nucleotides, revealing the induction of oxidative DNA fragmentation (Fig. 7B). The overproduction/accumulation of ROS can promote the damage of pentose and nucleotides [45, 46]. Due to the abstraction of a hydrogen atom from deoxyribose or inadequate repair of oxidized nitrogenous bases, especially 8-oxoguanine, ROS production enhances double-stranded DNA damage [45–47]. Herein, an increased level of fragmented DNA was observed in *P. aeruginosa* cells treated with Cu-phendione. Similarly, the treatment of *E. coli* with bactericidal antibiotics (β-lactams, fluoroquinolones and aminoglycosides) showed higher levels of DNA oxidation and fragmentation [48]. The treatment of *C. albicans* with Ag-phendione induced extensive smearing of DNA, indicating non-specific cleavage of the DNA [25]. In addition, several studies have reported that copper nanoparticles dramatically affect the bacterial redox systems that culminate with DNA fragmentation [49–51].

## Conclusions

The emergence of antimicrobial resistance is a severe public-health threat worldwide. It was reported that at least 700,000 people die annually from multidrug-resistant infections, and it was also estimated that the number of antimicrobial resistant-associated deaths could reach 10 million by 2050 [52]. The current work aims to provide an understanding of the interaction between phendione-derivative compounds and DNA, which can at least in part highlight the antimicrobial potential of Ag-phendione and Cu-phendione. Our group has been investigating the biological activity of Ag-phendione and Cu-phendione against both planktonic- and biofilm-growing of *P. aeruginosa* cells [9]. Herein, we showed that phendione-based compound, particularly Cu-phendione, were able to interact with double-stranded DNA and promote oxidative damage. In addition, Cu-phendione promoted damage in pseudomonal DNA. Reasons for this enhanced activity may stem from the superior DNA-binding affinity of Cu-phendione (*K*_app_ = 2.55 × 10^6^ M^−1^) compared to the parent [Cu(1,10-phen)_2_]^2+^ complex (*K*_app_ = 6.67 × 10^5^ M^−1^) [28]. Thus, it appears a combination of DNA binding and redox activity is required to achieve appropriate intracellular DNA cleavage. In vitro studies with pUC19 DNA can demonstrate cleavage activity by Cu(II) complexes—including those with moderate/low binding affinity—however, in more complex biological environments, the DNA-binding affinity of metal complexes becomes important and those with higher affinity (in particular polynuclear complexes) often produce significantly higher levels of intracellular DNA damage [37]. Altogether, we conclude that the antimicrobial activity of Cu-phendione could, in part, be correlated with the artificial metallo-nuclease activity of Cu-phendione. Still, Ag-phendione was not able to induce oxidative damage to DNA, indicating that this molecule might rely on other bactericidal mechanisms to kill *P. aeruginosa* cells. The potential application of phendione-derivative compounds as antimicrobial agents was supported by in vivo studies that showed that these compounds have non-mutagenic profile, and low toxicity in Swiss mice model (100% survival ≤ 150 mg/kg) [10, 17], which opens a new avenue in the search for biologically activity compounds especially against widespread and multidrug-resistant bacterial pathogens like *P. aeruginosa*.

## Supplementary Information

Below is the link to the electronic supplementary material.

Supplementary file1 (PDF 528 KB)
