# Supplementary material for: Copper(II) and silver(I)-1,10-phenanthroline-5,6-dione complexes interact with double-stranded DNA: further evidence of their apparent multi-modal activity towards Pseudomonas aeruginosa
Source: J Biol Inorg Chem. 2022 Jan 10;27(1):201–13. doi: 10.1007/s00775-021-01922-3 (PMC8840922; doi:10.1007/s00775-021-01922-3)
Supplement: Supplementary file 1 — Supplementary file1 (PDF 528 KB) [file 775_2021_1922_MOESM1_ESM.pdf]

Copper(II) and silver(I)-1,10-phenanthroline-5,6-dione complexes interact with double-stranded DNA: further evidence of their apparent multi-modal activity towards *Pseudomonas aeruginosa*

Anna Clara M. Galdino[1,2,#], Lívia Viganor[1,3,#], Matheus M. Pereira[4], Michael Devereux[3], Malachy McCann[5], Marta H. Branquinha[1], Zara Molphy[6,7], Sinéad O'Carroll[6], Conor Bain[6], Georgia Menounou[6,7], Andrew Kellett[6,7,*] and André L.S. Santos[1,2,*]

[1]Department of General Microbiology, Institute of Microbiology Paulo de Góes, Universidade Federal do Rio de Janeiro, Rio de Janeiro, Brazil; [2]Postgraduate Program in Biochemistry, Institute of Chemistry, Universidade Federal do Rio de Janeiro, Rio de Janeiro, Brazil; [3]The Centre for Biomimetic and Therapeutic Research, Focas Research Institute, Technological University Dublin, Dublin, Ireland; [4]CICECO - Aveiro Institute of Materials, Department of Chemistry, University of Aveiro, Portugal; [5]Chemistry Department, Maynooth University, Kildare, Ireland; [6]School of Chemical Sciences and the National Institute for Cellular Biotechnology, Dublin City University, Dublin, Ireland; [7]SSPC, the SFI Research Centre for Pharmaceuticals, School of Chemical Sciences, Dublin City University, Glasnevin, Dublin 9, Ireland

[#]These authors contributed equally to this work.

*These authors share senior authorship. Corresponding authors at andrew.kellett@dcu.ie (Andrew Kellett) and andre@micro.ufrj.br (André Santos)

SUPPORTING INFORMATION

Table S1. Docking affinity energy and interacting DNA major groove with ligands predicted by AutoDock vinna.

Compound	Affinity (kcal/mol)	Interacting nucleic acids	Type of interaction	From	To	Distance (Å)
1,10-Phen	-5.0	Adenosine5	Hydrogen Bond	Adenosine5	1,10-phen	2.13
1,10-Phen	-5.0	Adenosine5	Hydrogen Bond	Adenosine5	1,10-phen	2.88
1,10-Phen	-5.0	Guanosine4	Hydrophobic	Guanosine4	1,10-phen	4.93
1,10-Phen	-5.0	Guanosine4	Hydrophobic	Guanosine4	1,10-phen	4.47
1,10-Phen	-5.0	Guanosine4	Hydrophobic	Guanosine4	1,10-phen	5.13
1,10-Phen	-5.0	Adenosine5	Hydrophobic	Adenosine5	1,10-phen	5.00
Phendione	-5.3	Adenosine5	Hydrogen Bond	Adenosine5	Phendione	2.29
Phendione	-5.3	Adenosine5	Hydrogen Bond	Adenosine5	Phendione	2.41
Phendione	-5.3	Adenosine6	Hydrogen Bond	Adenosine6	Phendione	2.74
Phendione	-5.3	Adenosine17	Hydrogen Bond	Phendione	Adenosine17	3.65
Phendione	-5.3	Adenosine18	Hydrogen Bond	Adenosine18	Phendione	2.45
Phendione	-5.3	Thymine19	Hydrophobic	Thymine19	Phendione	3.86
Phendione	-5.3	Adenosine18	Hydrophobic	Adenosine18	Phendione	4.99
Phendione	-5.3	Adenosine18	Hydrophobic	Adenosine18	Phendione	5.93
Phendione	-5.3	Adenosine18	Hydrophobic	Adenosine18	Phendione	5.73
Phendione	-5.3	Thymine19	Hydrophobic	Thymine19	Phendione	5.97
Ag-phendione	-7.5	Adenosine5	Hydrogen Bond	Adenosine5	Ag-phendione	2.91
Ag-phendione	-7.5	Thymine19	Hydrophobic	Thymine19	Ag-phendione	3.79
Cu-phendione	-7.9	Adenosine6	Hydrogen Bond	Adenosine6	Cu-phendione	2.71
Cu-phendione	-7.9	Guanosine16	Electrostatic	Guanosine16	Cu-phendione	4.21
Cu-phendione	-7.9	Guanosine16	Electrostatic	Guanosine16	Cu-phendione	3.64
Cu-phendione	-7.9	Guanosine16	Hydrophobic	Guanosine16	Cu-phendione	5.38
Cu-phendione	-7.9	Guanosine16	Hydrophobic	Guanosine16	Cu-phendione	5.44

Table S2. Docking affinity energy and interacting DNA minor groove with ligands predicted by AutoDock vinna.

Compound	Affinity (kcal/mol)	Interacting nucleic acids	Type of interaction	From	To	Distance (Å)
1,10-Phen	-6.0	Thymine18	Hydrogen Bond	1,10-phen	Thymine18	3.73
		Thymine20			Thymine20	3.63
Phendione	-6.2	Adenosine6		Phendione	Adenosine6	3.64
		Thymine20			Thymine20	3.55
Ag-phendione	-7.1	Cytosine21		Ag-phendione	Cytosine21	3.37
		Guanosine22	Electrostatic	Guanosine22	Ag-phendione	4.40
Cu-phendione	-7.2	Thymine19	Hydrogen Bond	Thymine19	Cu-phendione	3.74
		Thymine18	Electrostatic	Thymine18		4.11
		Cytosine9		Cytosine9		4.56
		Thymine20		Thymine20		4.96
						4.35
		Cytosine21		Cytosine21		4.69
		Thymine20	Hydrophobic	Thymine20		3.90

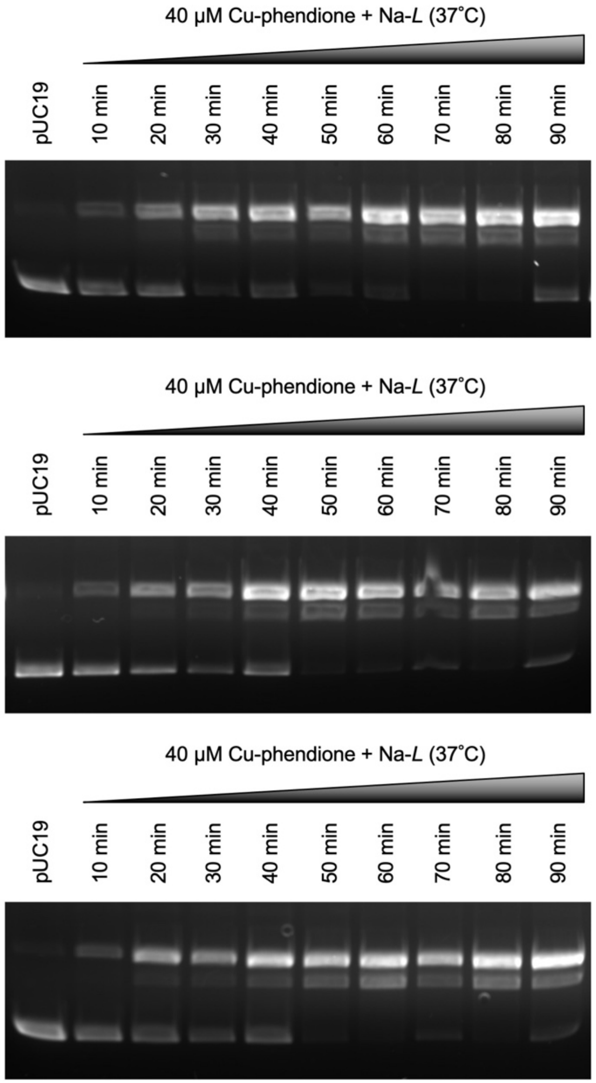

Figure S1: Triplicate kinetic study. 400 ng pUC19 plasmid DNA treated with 40 µM Cu-phendione every 10 min for 90 min at 37°C in the presence of 1 mM Na-*L*-Ascorbic (Na-L) and 25 mM NaCl. Band densitometry was carried out on the SynGene G:BOX mini6 using SynGene Gene Tools software.

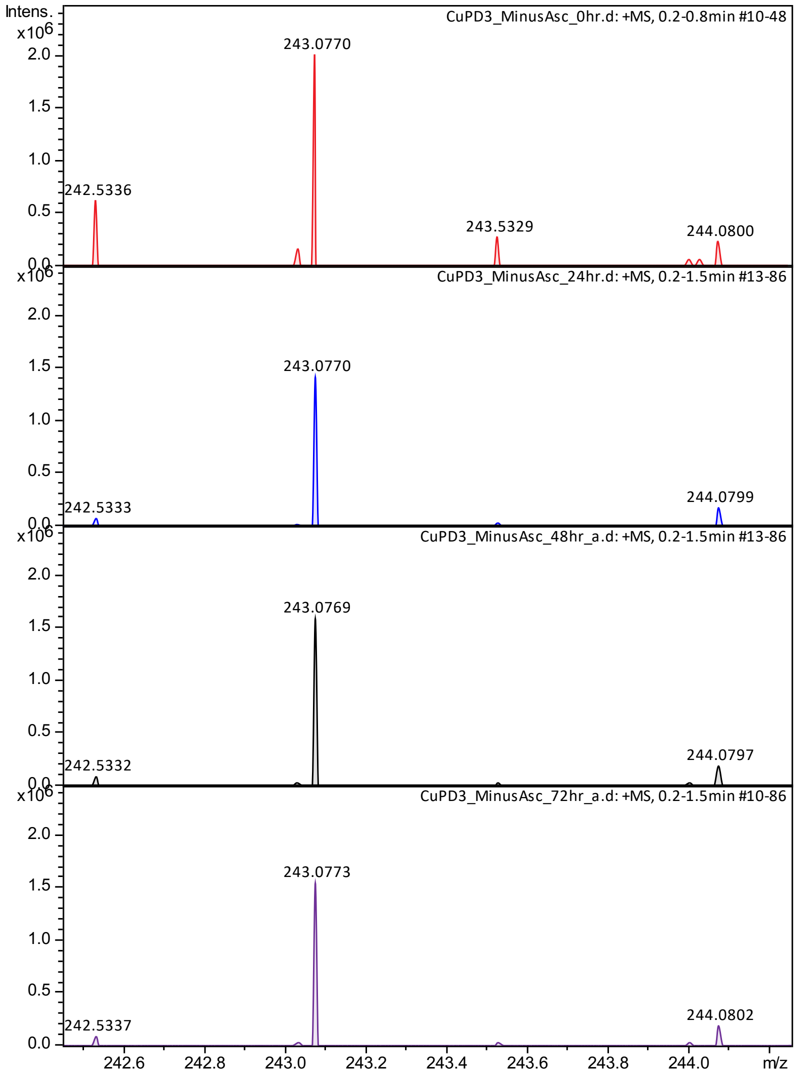

Figure S2: ESI-MS spectra of the $[Cu(phendione)_2]^{2+}$ complex recorded at 0, 24, 48, and 72 h. The complex was generated *in situ* by incubation of 1,10-phenanthroline-5,6-dione (phendione) with Cu(II) nitrate at a respective molar ratio of 3:1.

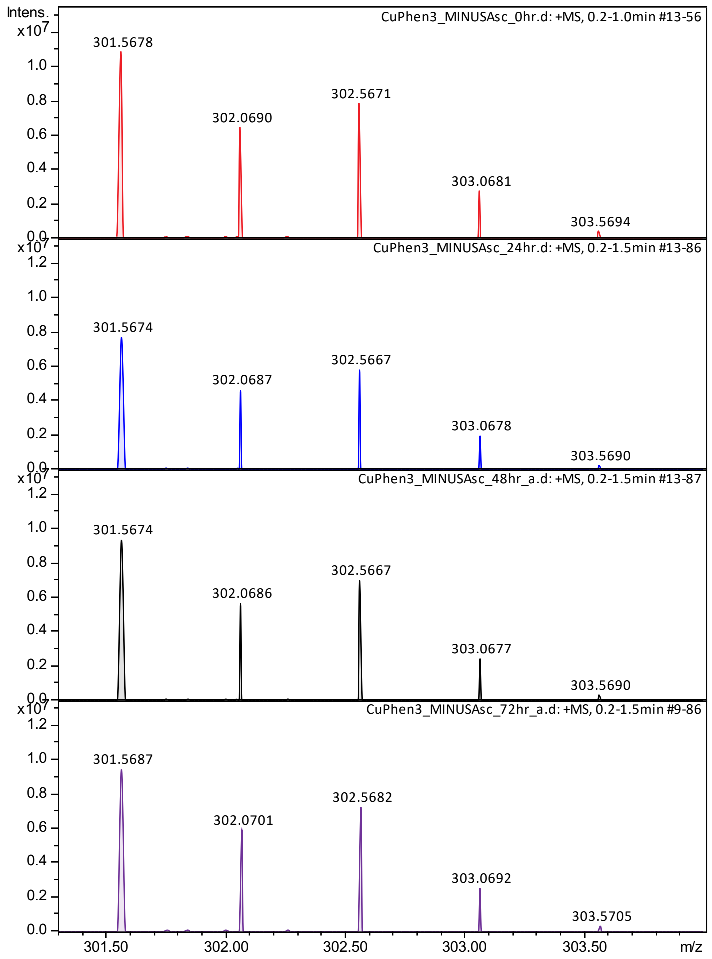

Figure S3: ESI-MS spectra of the $[Cu(phen)_3]^{2+}$ complex recorded at 0, 24, 48, and 72 h. The complex was generated *in situ* by incubation of 1,10-phenanthroline (phen) with Cu(II) nitrate at a respective molar ratio of 3:1.
